# Autosomal recessive woolly hair/hypotrichosis caused by LIPH mutations: a case report

**DOI:** 10.3389/fmed.2025.1563299

**Published:** 2025-07-02

**Authors:** Ying Xie, Sha Luo, Yumei Yang, Xin Zou, Shuying Lv, Meijiao Du, Yonglong Xu, Xiaojuan Song, Changjie Qi, Nuo Li, Dingquan Yang

**Affiliations:** ^1^Beijing University of Chinese Medicine, Beijing, China; ^2^Department of Dermatology, The National Center for the Integration of Traditional Chinese and Western Medicine, China-Japan Friendship Hospital, Beijing, China; ^3^Department of Dermatology, The First Affiliated Hospital of Hunan University of Chinese Medicine, Changsha, China; ^4^School of Senior Translation College, Dalian University of Foreign Languages, Dalian, China; ^5^Capital Medical University, Beijing, China; ^6^Department of Rehabilitation Medicine, The Eighth Medical Center, PLA General Hospital, Beijing, China

**Keywords:** woolly hair, autosomal recessive, LIPH gene, mutation, case report

## Abstract

**Objective:**

To report the clinical characteristics of a case of Autosomal recessive woolly hair/ hypotrichosis(ARWH/HT, OMIM:278150/604379) in a child, analyze and identify the causative gene and mutation site. A review of related research at home and abroad was conducted to summarize the current progress in the diagnosis and treatment of ARWH.

**Methods:**

Clinical data were collected, and exome sequencing was performed on blood samples from the patient and parents to screen for mutations. Sanger sequencing validated suspected pathogenic variants. A summary analysis of previously published woolly hair cases was also conducted.

**Results:**

The family was found to have mutations in the LIPH gene, with the patient’s sample showing two heterozygous mutations: *c.1101del* (maternal) and *c.736 T > A* (paternal). These compound heterozygous mutations are responsible for the ARWH phenotype.

**Conclusion:**

The compound heterozygous mutations *c.1101del* and *c.736 T > A* in the LIPH gene are the pathogenic mutations causing the clinical phenotype of autosomal recessive woolly hair in the child. The *c.1101del* mutation is a newly discovered frameshift mutation, enriching the mutation spectrum of LIPH-associated autosomal recessive woolly hair with hypotrichosis.

## 1 Introduction

Autosomal recessive woolly hair/ hypotrichosis(ARWH/HT, OMIM:278150/604379) is a rare congenital hair abnormality that usually manifests itself at birth or within the first two years of life (1). Clinically, it presents as sparse, thin, and tightly curled hair, resembling sheep’s wool, and may be accompanied by reduced hair pigmentation and increased hair fragility. Depending on its presentation and distribution, it can be classified as generalized or localized. Generalized woolly hair affects the entire scalp and extends over the entire body, while localized woolly hair is confined to a specific area of the scalp in the form of a woolly hair nevus. There is also a diffuse partial form that primarily occurs during adolescence and adulthood. This condition can occur alone or as part of a genetic syndrome, and can be divided into syndrome forms and non-syndrome forms (2).

In 1907, Gossage first observed and reported this phenomenon in a European family (3). In 1974, Hutchinson et al. classified non-syndromic woolly hair into three types based on genetic characteristics: ① Woolly hair nevus (non-hereditary); ② Autosomal dominant woolly hair (ADWH, hereditary); ③ Autosomal recessive woolly hair (ARWH, familial). It has been confirmed that non-syndromic autosomal recessive woolly hair is related to mutations in the C3ORF52, LIPH, LPAR6 (also known as P2RY5), and KRT25 genes, among which LIPH and LPAR6 gene mutations are the main causes (4). This article will report a case of autosomal recessive woolly hair, study the LIPH gene mutation, and conduct a literature review and systematic summary on the clinical manifestations, diagnosis, genetics, and treatment of ARWH, with the aim of providing insights for the clinical diagnosis and treatment of ARWH.

## 2 Clinical data

A 22-year-old female patient presented to our hospital on October 17, 2024. The patient’s hair was noted to be curly and burnt yellow one month after birth. Sparse hair growth was observed on both sides of the anterior fontanelle, while eyelashes and eyebrows appeared normal. The hair grew slowly, reaching a length of about 5 cm before ceasing further growth. The hair was notably sparse, curly, yellowish, rough without luster, prone to tangling and knotting, easily breakable, and difficult to comb through. The patient had a healthy past medical history. The patient is an only child, and both parents were healthy with normal hair, not related by close kinship, and there were no family members with similar diseases.

### 2.1 Physical examination

**Figure 1:**
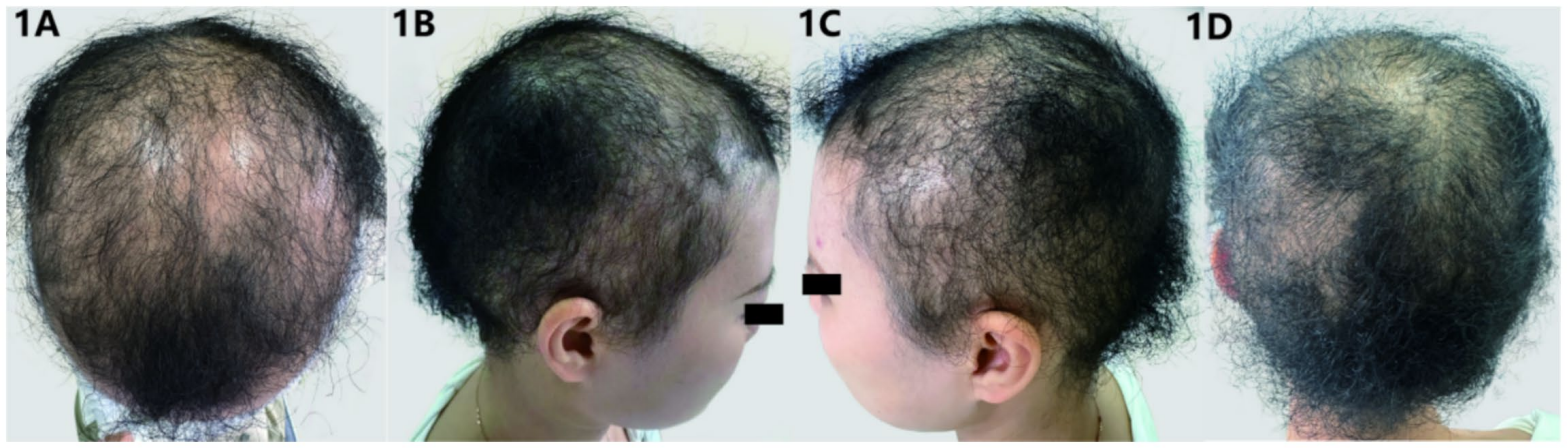
**(A–D)** Clinical manifestations: the patient’s hair is sparse, fine, and tightly curled, resembling lamb wool in appearance, with areas of exposed scalp visible.

The patient was generally well-nourished with normal development in intelligence and skeletal structure. Systemic examinations of the heart, lungs, and other systems revealed no abnormalities. There were no systemic abnormalities or unusual sweating. Dermatological findings included short, fine, soft, curly, fluffy hair with a lamb wool-like texture, light brown in color, resembling permed appearance, and diffusely sparse with some areas of scalp exposure. Hair brittleness increased, with a length of about 5 cm(Figure 1). The hair pull test was positive. Scalp skin color and texture were not significantly abnormal. Eyebrows, eyelashes, and body hair quantity and color showed no significant abnormalities. No obvious keratoderma was observed on palmoplantar areas, and teeth and nails were normal.

### 2.2 Blood examination

The patient’s blood routine, liver and kidney function, and thyroid function test results were all normal. Among the six sex hormones, estradiol, testosterone, and prolactin were normal, while progesterone, follicle - stimulating hormone (FSH), and luteinizing hormone (LH) were on the low side.

### 2.3 Trichoscopic examination

**Figure 2:**
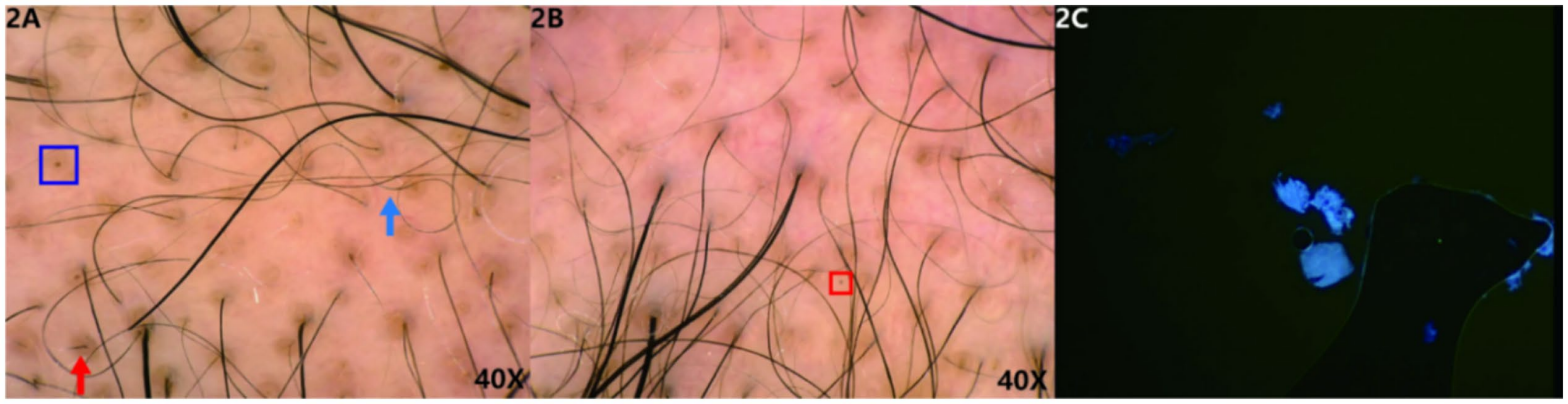
**(A,B)** Trichoscopic findings: some hair shafts appear wavy and curly (blue arrow), with signs of hair clumping, broken hair (red arrow), yellow dots (blue box), and black dots (red box). (Hongxin, polarised light, ×40). **2C** The patient’s fungal microscopy was negative.

Under trichoscopy, some hair shafts appeared wavy and curly, with signs of hair clumping, broken hair, yellow dots, and black dots (Figure 2). No exclamation mark hair or Phol-Pinkus narrowing was observed, and the scalp appeared essentially normal.

### 2.4 Microscopic examination

**Figure 3:**
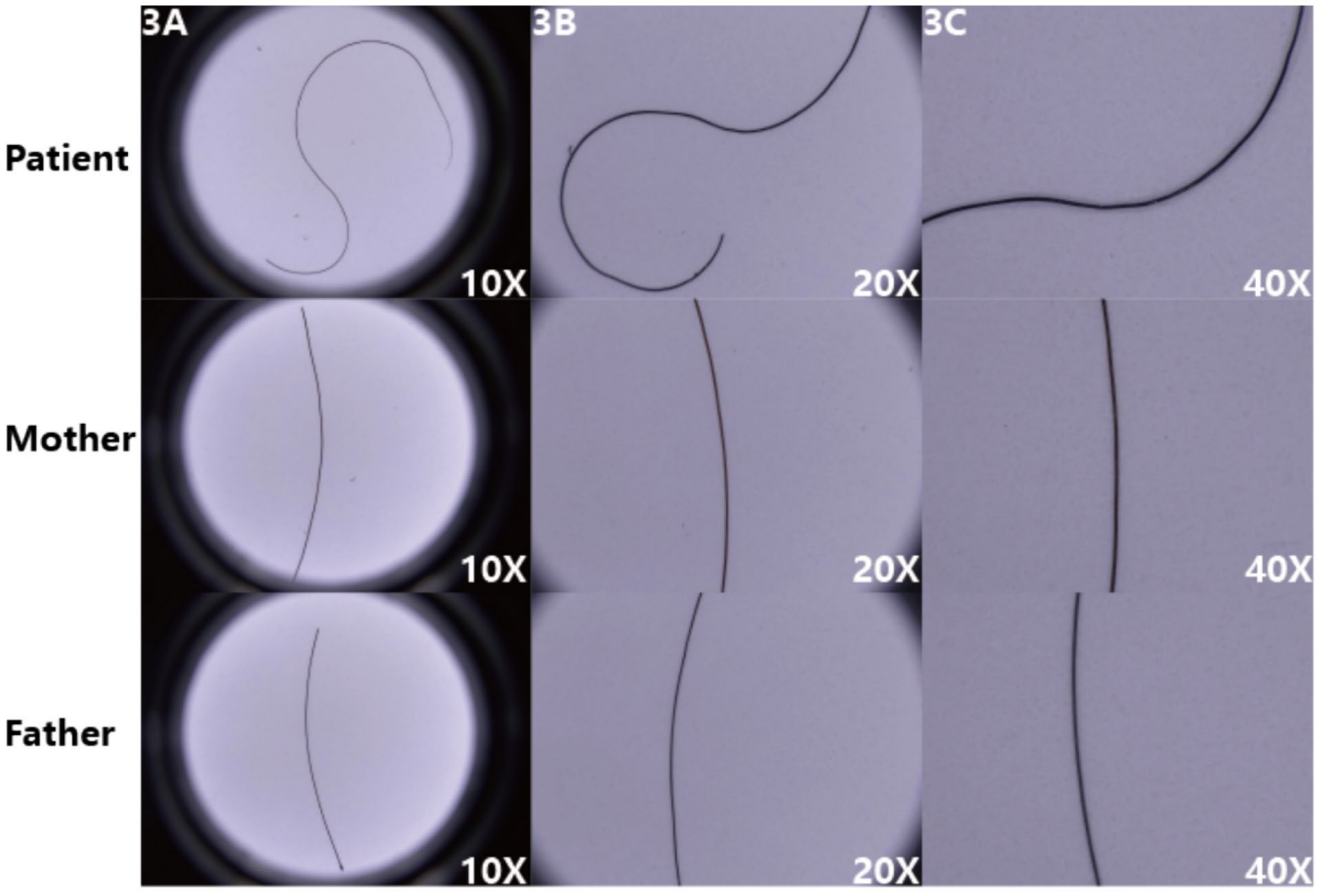
**(A–C)** The patient’s hair exhibits obvious irregular bending, whereas the parents’ hair appears relatively straight, intuitively reflecting the pathological characteristics of the patient’s hair.

The microscopic images (Figure 3) display the hair morphology of the patient and their parents at different magnifications (10X, 20X, 40X). The patient’s hair exhibits irregular bending, such as an “S”-shaped curve visible at 10X, and detailed abnormalities including local swelling at 20X and 40X, with a significantly disordered morphology. In contrast, the mother’s and father’s hair appears relatively straight at all magnifications, without obvious abnormal bending or morphological changes. The patient’s fungal microscopy was negative.

### 2.5 Scanning electron microscopy (SEM)

**Figure 4:**
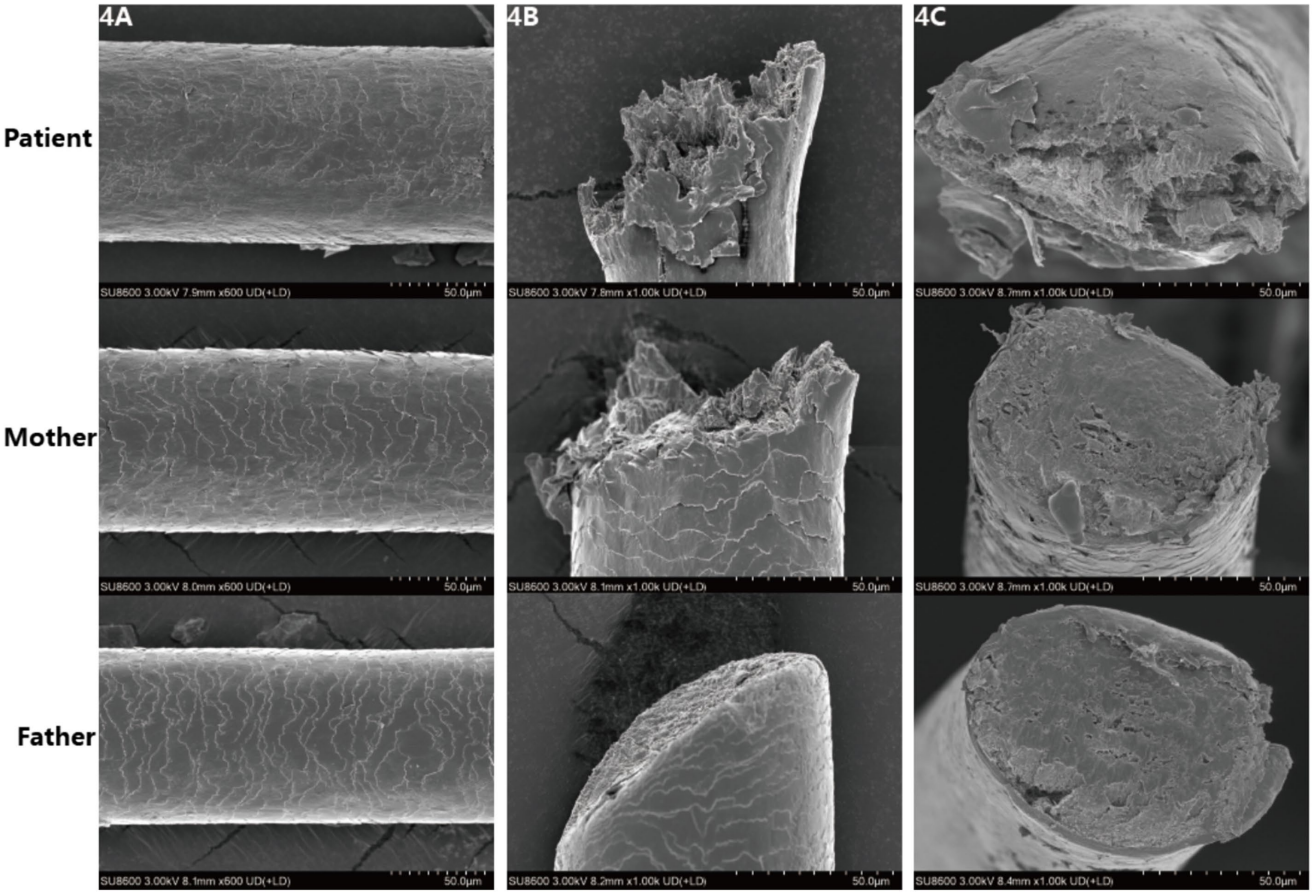
**(A–C)** Scanning electron microscopic images reveal irregular and rough cuticle structures in the patient’s affected hair, with oval-shaped cross sections.

SEM images showed (Figure 4) that the hair surface of the patient with pili torti presented obviously irregular textures, with numerous folds and grooves and a disordered structure. The cross-section of the hair was oval-shaped, with an extremely irregular internal structure, uneven edges, and suspected abnormalities in structures such as the cortex.

### 2.6 Treatment

Topical minoxidil tincture was administered to the patient. After approximately 3 months of use, follow-up showed no significant improvement, and the patient has not yet returned for a follow-up visit.

## 3 Genetic testing methods and results

### 3.1 Methods

After obtaining informed consent from the participant, peripheral blood samples were collected from the patient and their parents for analysis. DNA was fragmented and library preparation was performed. Target gene coding regions and adjacent splice sites were then captured and enriched using a chip-based method. Finally, high-throughput sequencing platforms were used to detect mutations.

## 4 Results

**Figure 5:**
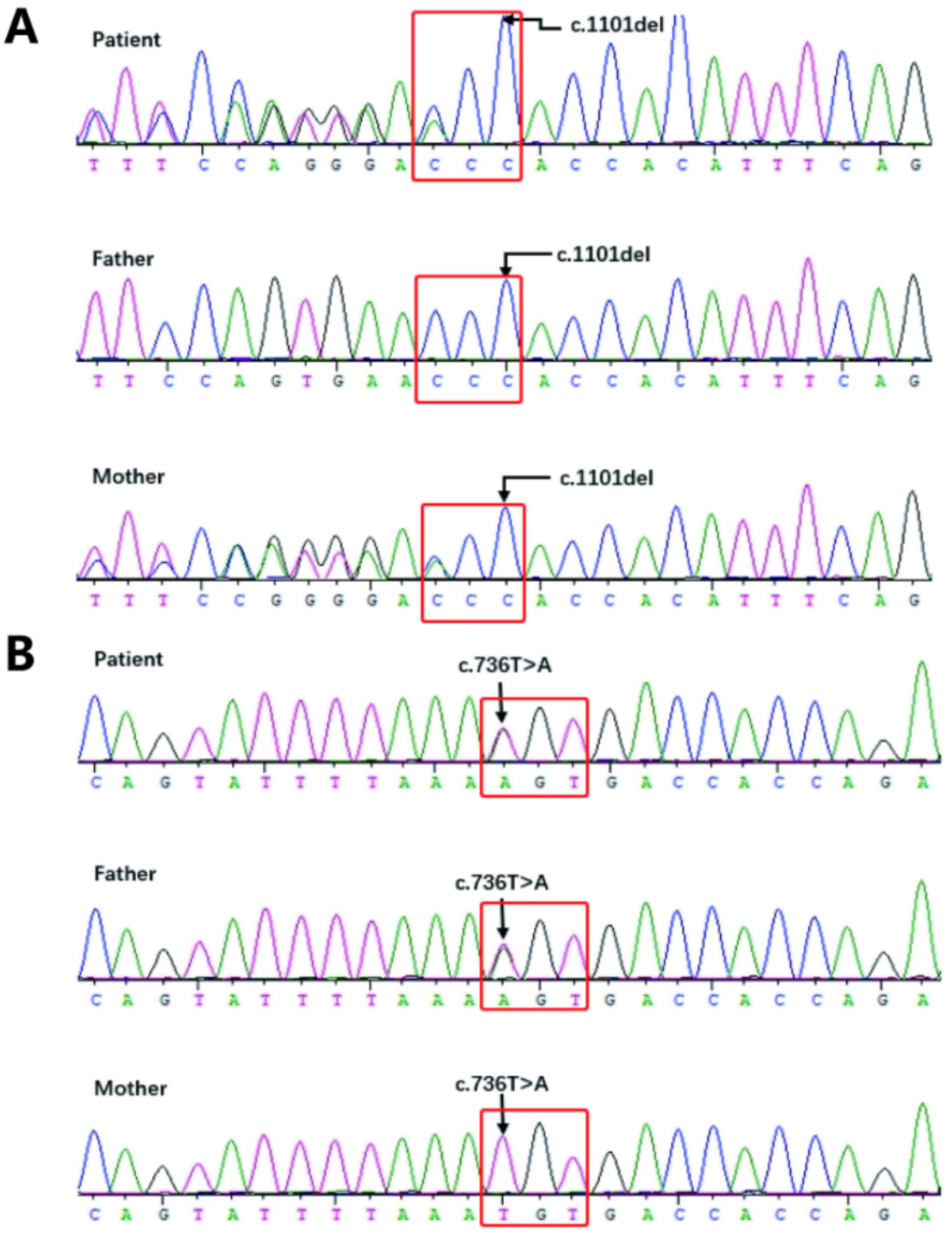
**(A,B)** Sanger Sequencing Validation Results for the Proband (Patient) and Their Parents: **5A:** The proband has a heterozygous missense mutation in exon 9, *c.1101del* (p. Thr368fs), while the mother also carries a heterozygous *c.1101del* (p. Thr368fs) mutation, and the father has no variation; **5B:** The proband has a heterozygous missense mutation in exon 6, *c.736 T > A* (p. Cys246Ser), while the father also carries a heterozygous *c.736 T > A* (p. Cys246Ser) mutation, and the mother has no variation.

The results showed that the patient’s sample had two heterozygous mutations in the LIPH gene: *c.1101del* and *c.736 T > A* (Figure 5). The *c.1101del* variant is located in exon 9, causing a frameshift mutation starting from amino acid position 368, leading to an early stop codon (p. Thr368fs). The *c.736 T > A* variant is located in exon 6, resulting in an amino acid change from cysteine to serine at position 246 (p. Cys246Ser). Family validation results showed that the *c.1101del* mutation was inherited from the mother, who has a heterozygous variation at this site, and the *c.736 T > A* mutation was inherited from the father, who also has a heterozygous variation at this site, consistent with autosomal recessive inheritance patterns. According to the ACMG guidelines, both mutations are classified as pathogenic. The frameshift mutation *c.1101del* has not been previously reported and is considered a novel variant. Genetic counseling has been provided to the patient.

## 5 Discussion

This paper reports a novel case of autosomal recessive woolly hair (ARWH) with compound heterozygous LIPH mutations (*c.1101del* and *c.736 T > A*), including the first reported frameshift variant *c.1101del*. This finding expands the mutational spectrum of ARWH and reinforces genotype–phenotype correlations in hereditary hair disorders. The patient exhibited classic non-syndromic ARWH features: congenital tightly coiled “sheep wool-like” hair with preserved eyebrows, sweat glands, and nails, consistent with prior reports (3).

With the development of molecular biology experimental techniques, the pathogenic genes of ARWH have been mapped and cloned through two-point linkage analysis. Type 1 autosomal recessive woolly hair (ARWH1) is caused by mutations in the LPAR6 (also known as P2RY5) gene. Type 2 autosomal recessive woolly hair (ARWH2) is caused by mutations in the LIPH gene. Type 3 autosomal recessive woolly hair (ARWH3) is caused by mutations in the KRT25 gene. Additionally, in recent years, missense mutations in the C3ORF52 gene have been observed in ARWH patients from two independent families (3).

The groundbreaking discovery by Kazantseva’s team in 2006 linking LIPH mutations to hair developmental defects laid a critical foundation for understanding genetic hair disorders. To date, over 30 pathogenic LIPH variants have been identified across global populations, underscoring the gene’s pivotal role in ARWH2. Located at 3q27.2, this gene encodes a membrane-bound triglyceride lipase that catalyzes phosphatidic acid (PA) hydrolysis to produce lysophosphatidic acid (LPA)—a bioactive lipid essential for hair follicle development through activation of the P2Y5 receptor. The spatial co-expression of LIPH and P2Y5 in specific anatomical layers of the hair follicle inner root sheath (IRS), particularly the Huxley and basement membrane layers, provides histological validation of the LIPH/LPA/P2Y5 signaling axis as a central regulator of follicular differentiation and maturation (5).

In this case, the novel *c.1101del* frameshift mutation (p. Thr368Profs*29) causes premature termination of LIPH’s catalytic domain, predicted to impair PA hydrolytic activity. While bioinformatic analyses strongly support its pathogenicity, the quantitative impact of this truncation on LPA-P2Y5 signaling dynamics remains to be fully elucidated.

The treatment of ARWH is rather challenging. With the gradual progress of research on the LIPH/LPA/P2Y5 signaling pathway and the pathogenesis of ARWH, the development of targeted drugs that regulate this signaling pathway may potentially become a treatment method for this disease. However, further in-depth genetic and pathophysiological research is still required to improve the diagnostic and treatment protocols, promote the development of diagnostic and treatment strategies for ARWH, and enhance the quality of life of patients.

## Data Availability

The original contributions presented in the study are included in the article/supplementary material, further inquiries can be directed to the corresponding author/s.
