# Correction: Autosomal recessive woolly hair/hypotrichosis caused by LIPH mutations: a case report

**DOI:** 10.3389/fmed.2025.1677113

**Published:** 2025-08-21

**Authors:** Ying Xie, Sha Luo, Yumei Yang, Xin Zou, Shuying Lv, Meijiao Du, Yonglong Xu, Xiaojuan Song, Changjie Qi, Nuo Li, Dingquan Yang

**Affiliations:** ^1^Beijing University of Chinese Medicine, Beijing, China; ^2^Department of Dermatology, The National Center for the Integration of Traditional Chinese and Western Medicine, China-Japan Friendship Hospital, Beijing, China; ^3^Department of Dermatology, The First Affiliated Hospital of Hunan University of Chinese Medicine, Changsha, China; ^4^School of Senior Translation College, Dalian University of Foreign Languages, Dalian, China; ^5^Capital Medical University, Beijing, China; ^6^Department of Rehabilitation Medicine, The Eighth Medical Center, PLA General Hospital, Beijing, China

**Keywords:** woolly hair, autosomal recessive, LIPH gene, mutation, case report

The funder China-Japan Friendship Hospital, Grant Number 2024-HX-24 was erroneously omitted. The corrected funding statement appears below:

“The author(s) declare that financial support was received for the research and/or publication of this article. This work was supported by China-Japan Friendship Hospital, Grant Number 2024-HX-24 to all authors.”

The original version of this article has been updated.

